# Do preexisting antibodies against seasonal coronaviruses have a protective role against SARS-CoV-2 infections and impact on COVID-19 severity?

**DOI:** 10.1016/j.ebiom.2022.103831

**Published:** 2022-01-23

**Authors:** Gheyath K. Nasrallah

**Affiliations:** a Member of QU Health, Department of Biomedical Science, College of Health Sciences, Qatar University, P.O. Box 2713, Doha, Qatar; b Biomedical Research Center, Qatar University, Doha, Qatar

**Keywords:** sCoV, Preexisting immunity, SARS-CoV-2, Cross-protectivity

Because of the emergence of coronavirus disease 2019 (COVID-19), caused by the novel severe acute respiratory syndrome coronavirus (SARS-CoV-2), many questions remain unresolved regarding the abundance of cross-reactivity between the SARS-CoV-2 and other human seasonal coronaviruses (sCoVs) antigens and the role of the sCoVs preexisting antibodies in protective immunity to SARS-CoV-2.

Four endemic human sCoVs (NL63, 229E, OC43, and HKU1), which cause the common cold and recurrent respiratory disease, are highly prevalent worldwide. While almost everyone has been exposed to at least one of these sCoVs, immune response to each sCoV declines over time.^1^ These human sCoVs share striking sequence similarities with the E-envelope (96%), M-membrane (91%), and N-nucleocapsid (91%) proteins of SARS-CoV-2.^2^ However, they only share about 24–30% similarities with the trimeric spike S-protein (S-trimer). The S-protein is considered the major target protein/antigen for the protective humoral and cellular immunity. That is, the S-protein contains the angiotensin-converting enzyme 2 (ACE2) receptor binding domain (known as S-RBD) that is important for viral cell entry.^2^ Due to the apparent similarities between sCoVs, cross-reactivity between antibodies elicited by different sCoVs and cognate antibodies targeting SARS-CoV-2 antigens is expected.^2^

The protective role of preexisting cellular and humoral immunity (cross-reactive antibodies) from exposure to sCoVs against SARS-CoV-2 infections is controversial. Some studies reported that sCoV antibodies are boosted upon SARS-CoV-2 infection but not associated with protection,^3^ while others provided various lines of evidence for preexisting humoral and cellular cross-neutralization and protection against SARS-CoV-2.^4^ To investigate the role the humoral immune response in cross-protection between sCoVs and SARS-CoV-2, Galipeau et al., conducted a cross-sectional study to determine the level of cross-reactivity and cross-neutralization to three SARS-CoV-2 antigens (S-RBD, S-trimer, N) in pre-pandemic serum samples.^5^ The samples were collected from four different study groups, pediatrics and young adults (> 21), adults (21–70 years), elders (> 70 years), and patients infected with HCV or HIV (control group). 580 pre-pandemic samples and 178 post-pandemic samples were collected from diverse sources, including Icahn School of Medicine at Mount Sinai, Eastern Ontario Regional Laboratory Association (EORLA), and Ottawa Hospital (TOH). This study reported a relatively high level of cross-reactivity against various SARS-CoV-2 epitopes, with N being the most frequently detected (11%), followed by S-trimer (5%). These results align with a previous study that reported 16.2% cross-reactivity to N and 4.2% for S-trimer.^6^ The authors concluded that although N-antibodies are unlikely to be neutralizing, the Fc region of the N-antibodies may elicit strong effector functions through different protective mechanisms such as antibody-dependent cell mediated cytotoxicity (ADCC), complement activation, and priming the CD8+ T*-*cell responses, which may indirectly influence other antiviral pathways in the infected patients.^7^

With the measurement of different antibody isotypes and subclasses against the sCoV antigens, Galipeau and his colleagues demonstrated that pre-pandemic samples exhibit immunoreactivity to SARS-CoV-2 protein/antigens. Although there was no direct association between the titer of sCoV antibodies and neutralization, there was a significant predictive correlation between neutralization of S binding and the relative ratios of the different sCoV antibodies, with NL63 and OC43 being the most weighted for this prediction. These findings provide credence to the hypothesis that latent factors linked with sCoV exposure have a predictive and protective role against SARS-CoV-2 and potentially impact the disease severity. Further, using machine learning procedures, the authors demonstrated that the neutralizing ability of these antibodies to block RBD/ACE2 binding depends on relative ratios of IgGs directed to all four sCoV spike antigens. The strength of this study stems from including a large sample size and extensively characterizing the cross-reactive humoral immune response by covering all SARS-CoV-2 and the four sCoVs structural proteins using different serological assays.

The findings of Galipeau et al. study are three-fold; First, it is not the absolute levels of sCoVs antibodies that are predictive of neutralization but rather the relative ratios to all sCoVs. Second, it is the first study to demonstrate a functional relationship between prior exposure to sCoVs and neutralization of SARS-CoV-2 S-RBD by cross-reactive antibodies. Third, the ability to accurately predict which individuals can neutralize SARS-CoV-2 spike-ACE2 interactions using *in silico* methods such as Machine Learning. It is worth noting that preexisting memory T cells induced by sCoVs can shape susceptibility to and the clinical severity of SARS-CoV-2.^8^ Therefore, together with preexisting B-cell and T-cell memory, the relative ratios of all antibodies against sCoVs may substantially reduce viral transmission and mitigate the severity of the symptoms. Nevertheless, it is essential to determine the extent, positive or negative, to which the humoral immune response to SARS-CoV-2 contributes to virus-induced immunopathogenesis.

## CRediT authorship contribution statement

**Gheyath K. Nasrallah:** Conceptualization, Writing – review & editing.

## Declaration of interests

The author declares no conflict of interest.

## References

[1] Edridge A.W.D., Kaczorowska J., Hoste A.C.R. (2020). Seasonal coronavirus protective immunity is short-lasting. Nat Med.

[2] Bates T.A., Weinstein J.B., Farley S., Leier H.C., Messer W.B., Tafesse F.G. (2021). Cross-reactivity of SARS-CoV structural protein antibodies against SARS-CoV-2. Cell Rep.

[3] Anderson E.M., Goodwin E.C., Verma A. (2021). Seasonal human coronavirus antibodies are boosted upon SARS-CoV-2 infection but not associated with protection. Cell.

[4] Sagar M., Reifler K., Rossi M. (2021). Recent endemic coronavirus infection is associated with less-severe COVID-19. J Clin Invest.

[5] Galipeau Y., Siragam V., Laroche G. (2021). Relative ratios of human seasonal coronavirus antibodies predict the efficiency of cross-neutralization of SARS-CoV-2 spike binding to ACE2. EBioMedicine.

[6] Hicks J., Klumpp-Thomas C., Kalish H. (2021). Serologic cross-reactivity of SARS-CoV-2 with endemic and seasonal Betacoronaviruses. J Clin Immunol.

[7] Morgenlander W.R., Henson S.N., Monaco D.R. (2021). Antibody responses to endemic coronaviruses modulate COVID-19 convalescent plasma functionality. J Clin Invest.

[8] Sette A., Crotty S. (2020). Preexisting immunity to SARS-CoV-2: the knowns and unknowns. Nat Rev Immunol.

